# Characteristics of Carbapenem-resistant Klebsiella pneumoniae in sewage from a tertiary hospital in Jilin Province, China

**DOI:** 10.1371/journal.pone.0285730

**Published:** 2023-05-17

**Authors:** Mingwei Liu, Lin Zheng, Lingwei Zhu, Gejin Lu, Hongru Guo, Jiayao Guan, Jie Jing, Shiwen Sun, Ying Wang, Zixian Wang, Yang Sun, Xue Ji, Bowen Jiang, Jun Liu, Wenhui Zhang, Xuejun Guo

**Affiliations:** 1 College of Life Science, Jilin Agricultural University, Changchun, China; 2 Key Laboratory of Jilin Province for Zoonosis Prevention and Control, Changchun Veterinary Research Institute, Chinese Academy of Agricultural Sciences, Changchun, China; 3 The Sericultural Research Institute of Jilin Province, Jilin, PR China; Tribhuvan University, NEPAL

## Abstract

Carbapenem-resistant *Klebsiella pneumoniae* (CRKP) infection is a serious problem in hospitals worldwide. We monitored a tertiary hospital in Changchun, Jilin Province, China, and found that CRKP was the major species among the carbapenem-resistant isolates in sewage. Subsequently, we evaluated the drug susceptibility, resistance genes, virulence genes, outer pore membrane protein-related genes (*OmpK35 & OmpK 36*), multi-locus sequence typing and replicons, biofilm formation capabilities, and resistance to chlorine-containing disinfectants among KP isolates. Identification of drug sensitivity, multiple resistance profiles were observed including 77 (82.80%) multidrug resistant (MDR), 16 (17.20%) extensive drug resistant (XDR). Some antibiotic resistance genes were detected, the most prevalent carbapenemase gene was *bla*_KPC_, and 16 resistance genes were associated with other antibiotics. In addition, 3 (3.23%) CRKP isolates demonstrated loss of OmpK-35 and 2 (2.15%) demonstrated loss of OmpK-36. In the detection of multi-locus sequence typing (MLST), 11 ST11 isolates carried virulence genes. The most common replicon type was IncFII. Biofilm-forming capabilities were demonstrated by 68.8% of the isolates, all of which were resistant to chlorine-containing disinfectants. The results of the study showed that antibiotic-resistant isolates, especially CRKP, could resist disinfectants in hospital wastewater, and improper treatment of hospital wastewater may lead to the spread of drug-resistant bacteria and their genes. Thus, these bacteria must be eliminated before being discharged into the municipal sewage system.

## Introduction

*Klebsiella pneumoniae* (KP) is a member of the ESKAPEE (*Enterococcus faecium*, *Staphylococcus aureus*, *Klebsiella pneumoniae*, *Acinetobacter baumannii*, *Pseudomonas aeruginosa*, *Enterobacter spp*, and *Escherichia coli*) family organisms [1]. Among these organisms, carbapenem-resistant *Klebsiella pneumoniae* (CRKP) has become the most common carbapenemase-producing species in hospitals [2] and is a major healthcare-associated pathogen worldwide. Research has shown that the incidence of CRKP infections and associated deaths increased 6.16 times from 2007–2015 [3]. KP is intrinsically resistant to multiple antibiotics and can easily acquire antibiotic resistance determinants. Furthermore, KP has the potential to evolve into multidrug resistance phenotypes and hypervirulent KP (HVKP). The ST11 KP strain is associated with a high drug resistance phenotype and high virulence [4].

Carbapenem antibiotics are the most effective against multidrug-resistant (MDR) gram-negative bacteria [5] and are used as a last resort to treat critical patients. The most common mechanism by which bacteria develop resistance to carbapenem antibiotics is the production of carbapenemases. KPC-2 and NDM are dominant carbapenemases observed in clinical CRKP cases in China. Pathogens producing KPC-2 are resistant to all β-lactams except ceftazidime–avibactam, greatly limiting treatment options; additionally, NDM is a serious threat to public health worldwide due to the rapid spread of NDM-expressing pathogens [6].

Hospitals play an important role in spreading antibiotic-resistant bacteria into the environment through sewage. Hospital sewage contains various antibiotic compounds, microorganisms, metabolized drugs, disinfectants, and patient excreta [7]. Thus, hospital sewage is a key reservoir for MDR microorganisms [8]. Hospital sewage analysis has emerged as a supplement to traditional clinical bacterial resistance monitoring because a single sewage sample contains bacteria from thousands of individuals [9].

In this study, we characterized the CRKP isolates in sewage from a tertiary hospital in Changchun City located in northeast China, confirmed the antibiotic resistance patterns and detection of some drug resistance genes, evaluated biofilm formation ability, and virulence genes, and performed multi-locus sequence typing (MLST).

## Materials and methods

### Sample collection and bacterial isolation

We selected a comprehensive tertiary hospital with over 5,900 beds and over 9,600 employees. It is one of the top 30 hospitals in China and is located in Changchun in the northeast Jilin province. The hospital has a standard sewage treatment system.

The samples were taken from sewage treatment stations and had not been subjected to digestion treatment. We used a 500-mL stainless steel barrel sampler to collect 200-mL sewage every two months four times from March to September 2021. The sampling procedure was approved by Changchun Veterinary Research Institute, Chinese Academy of Agricultural Sciences. The samples were stored on ice, transported to the laboratory, and processed within 6 h at 4°C. One hundred microliters of each collected sewage sample was spread on MacConkey agar (Haibo Biotech, Qingdao, China) plates containing 4 mg/L imipenem and 2 mg/L vancomycin (to inhibit gram-positive bacteria) and incubated for 20 h at 37°C. The 16S rDNA gene was amplified and sequenced by the Comate Biosciences Co., Ltd. (Changchun, China), and biochemical identificationwas determined using a BD Phoenix^TM^-100 System.

### String testing and virulence gene detection

The hypermucoviscous phenotype was identified by string test. All isolates were inoculated on agar plates containing 5% sheep blood and incubated at 37°C for 20 h. The string test was performed as previously described [10]. Virulence genes *rmpA*, *rmpA2*, *iucA*, and *iroB* were amplified by PCR as previously described [11].

### Multi-locus sequence typing

MLST was performed on all isolates by PCR of seven standard housekeeping genes (*gapA*, *infB*, *mdh*, *pgi*, *phoE*, *rpoB*, and *tonB*) listed on the PubMLST website (http://www.pasteur.fr/recherche/genopole/PF8/mlst/Kpneumoniae.html). The sequence types (STs) were determined using the MLST database.

### Antimicrobial susceptibility testing

The susceptibility of the isolates to amikacin, gentamicin, imipenem, meropenem, cefazolin, ceftazidime, cefotaxime, cefepime, aztreonam, ampicillin, piperacillin, amoxicillin–clavulanate, ampicillin–sulbactam, piperacillin–tazobactam, colistin, trimethoprim–sulfamethoxazole, chloramphenicol, ciprofloxacin, levofloxacin, moxifloxacin, and tetracycline was determined using a BD Phoenix^TM^-100 System. The minimum inhibitory concentration (MIC) of imipenem was determined using the broth microdilution method according to 2020 Clinical and Laboratory Standards Institute (CLSI). *E*. *coli* ATCC 25922 was used as the quality control for the susceptibility test. The isolates found to be resistant to at least three different classes of antimicrobial agents were classified as MDR bacteria, and resistant to at least one agent in all but two or fewer antimicrobial agents were classified as XDR bacteria [12].

### Detection of antimicrobial resistance genes

The presence of several drug resistance-associated genes was assessed by conventional single PCR. The template DNA consisted of boiled lysates prepared from the isolates. Carbapenem resistance genes (*bla*_KPC_, *bla*_NDM_, *bla*_IMP_, *bla*_VIM_, and *bla*_OXA–48_), β-lactam resistance genes (*bla*_CTX–M_, *bla*_SHV,_ *bla*_TEM_), plasmid-mediated quinolone resistance (PMQR) genes (*aac(6′)-Ib-cr*, *qnrA*, *qnrB*, *qnrC*, *qnrD*, and *qnrS*), tetracycline resistance genes (*tet(A)*, *tet(B)*, *tet(C)*, *tet(D)*, and *tet(X)*), sulfonamide resistance genes (*sul1*, *sul2*, *sul3*), chloramphenicol resistance genes *cmlA*, *floR*, aminoglycoside resistance genes (*armA*, *rmtB*, *aac(3)-II*, *aac(6′)-Ib*) and integrase genes intI1 (for class 1 integrons), intI2 (for class 2 integrons), and intI3 (for class 3 integrons) were evaluated. Furthermore, a single PCR assay targeting two outer membrane pore protein genes (*OmpK-35* and *OmpK3*6) was also performed [13]. The PCR primers and conditions are shown in S1 Table.

### Replicon typing

A PBRT kit (DIATHEVA, Italy) was used to identify the 25 different replicons (HI1, HI2, I1, I2, X1, X2, L/M, N, FIA, FIB, FIC, FII, FIIS, FIIK, W, Y, P, A/C, T, K, U, R, B/O, HIB-M, and FIB-M) in eight multiplex PCRs following the manufacturer’s instructions [14]. PCR products were visualized using QuickGel 6100 system (Monad Biotech CO., Ltd.)

### Disinfectant resistance test

Method for determining the MIC value in CLSI, the MIC value of the disinfectant NaClO was determined by micro-broth dilution. The concentration gradients were set as 1600 μg/ml, 800 μg/ml, 400 μg/ml, 200 μg/ml, 100 μg/ml, 50 μg/ml, and 25 μg/ml. *E*. *coli* ATCC 25922 was used as the quality control. Because the effective concentration of NaClO solution reported in the manufacturer’s instructions was 300–500 μg/ml, an isolate with a MIC value greater than or equal to 400 μg/ml was defined as resistant in this research.

### Biofilm formation assay

We modified routine methods to determine the biofilm formation capability of the isolates [15]. Briefly, the isolate to be tested was cultured in Mueller-Hinton broth (bioMérieux, Marcy-l’Étoile, France) at 37°C for 20 h; then the concentration was adjusted to 0.5 McFarland, the suspension was diluted 100 times, and 200 μL of the resulting suspension was inoculated into a sterile flat-bottomed 96-well polystyrene microplate. After a 20-h incubation at 37°C, the non-adherent cells were removed, and the plate was washed three times with 300 μl of 1% sterile phosphate-buffered saline (PBS) per well. Biofilms were fixed with methanol for 15 min, the remaining methanol was removed, and the well was air-dried. Subsequently, 220 μl 1% crystal violet was added per well and incubated for 10 min. The well was then rinsed with PBS, air dried, and 200 μl 75% ethanol was used to dissolve the dyed biofilm. Finally, the optical density (OD) of the dissolved biofilm was measured with a microplate reader at a wavelength of 460 nm. Three repeat tests were conducted for each strain. Sterile Mueller-Hinton Broth medium (Solarbio, Beijing, China) was used as a negative control. The cut-off for biofilm formation was defined as three standard deviations above the mean of the negative control OD (ODc). The criteria were as follows: OD <ODc, no biofilm forming ability; ODc<OD<2ODc, weak biofilm forming ability; 2ODc<OD<4ODc, moderate biofilm forming ability, and 4ODc< ODc, strong biofilm formation ability.

### Statistical analyses

Chi-square test scores were used to assess statistically significant differences in biofilm formation ability and antibiotic resistance. All statistical analyses were performed using IBM SPSS Statistics for Windows version 26.0. International Business Machines Corporation, Armonk. A probability (p) value < 0.05 was considered statistically significant.

## Results

### Bacterial Isolation and Identification

**Fig 1:**
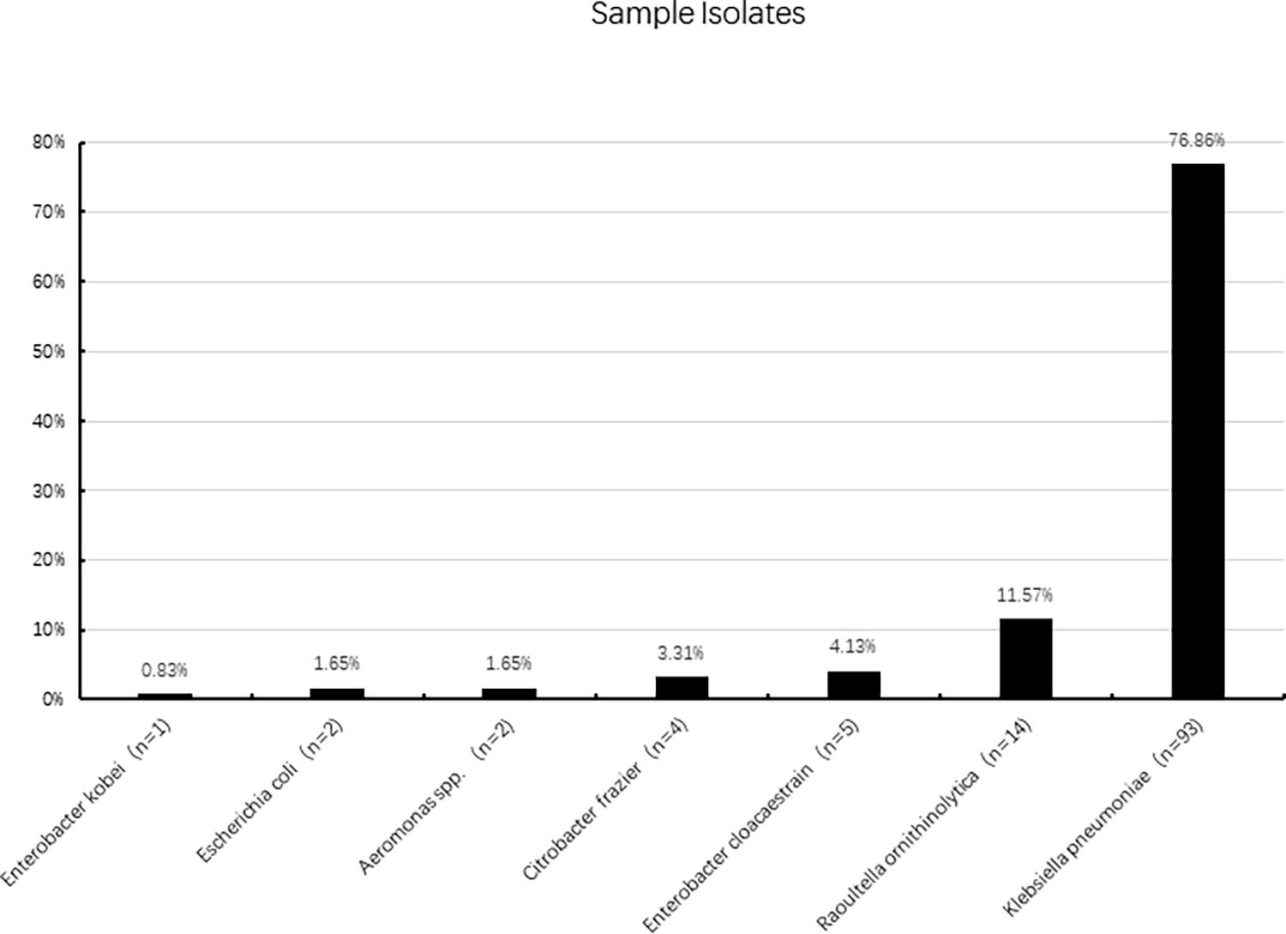
Distribution of 121 carbapenem-resistant isolates.

A total of 121 carbapenem-resistant isolates were isolated from four hospital sewage samples, including 1 strain of *Enterobacter kobei* (0.83%), 2 isolates of *E*. *coli* (1.65%), 4 isolates of *Citrobacter frazier (*3.31%), 14 isolates of strain of *Raoultella ornithinolytica (*11.57%), 2 isolates of *Aeromonas spp*. (1.65%), 5 isolates of *Enterobacter cloacaestrain (*4.13%) and 93 isolates of *Klebsiella pneumoniae (*76.86%) (Fig 1). All isolates were confirmed by 16S rDNA gene analysis and the BD Phoenix^TM^-100 System. Since KP accounted for the majority of the isolates, we subsequently focused on the characteristics of KP isolates. Among the 93 KP isolates, 2 were isolated in March 29 May, 23 in July, and 39 in September.

### Determination of antibiotic susceptibility of the isolated CRKP

**Fig 2:**
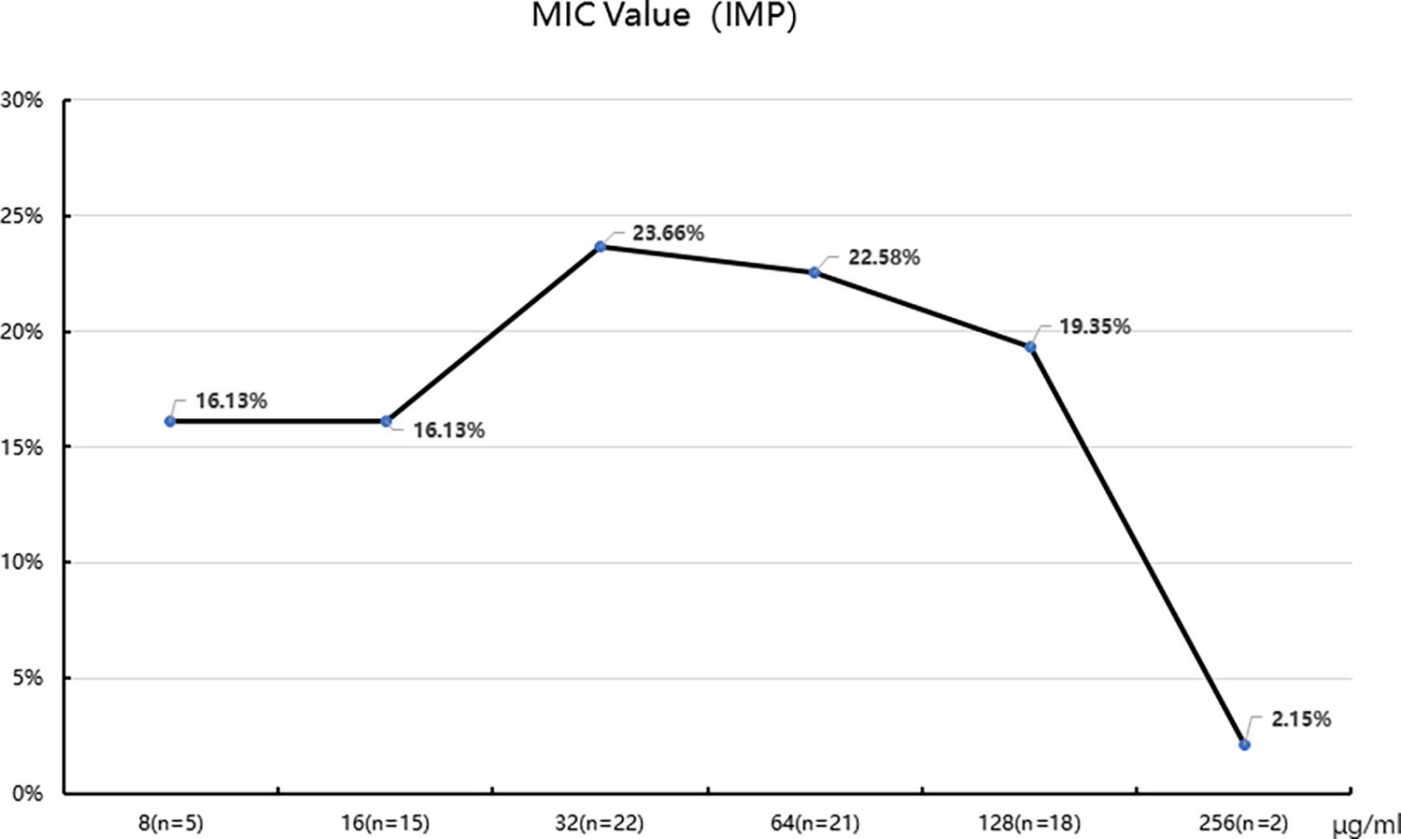
MIC of 93 *Klebsiella pneumoniae* isolates against imipenem. Note. Rows represent imipenem concentration gradients, and columns represent percentages of isolates.

**Fig 3:**
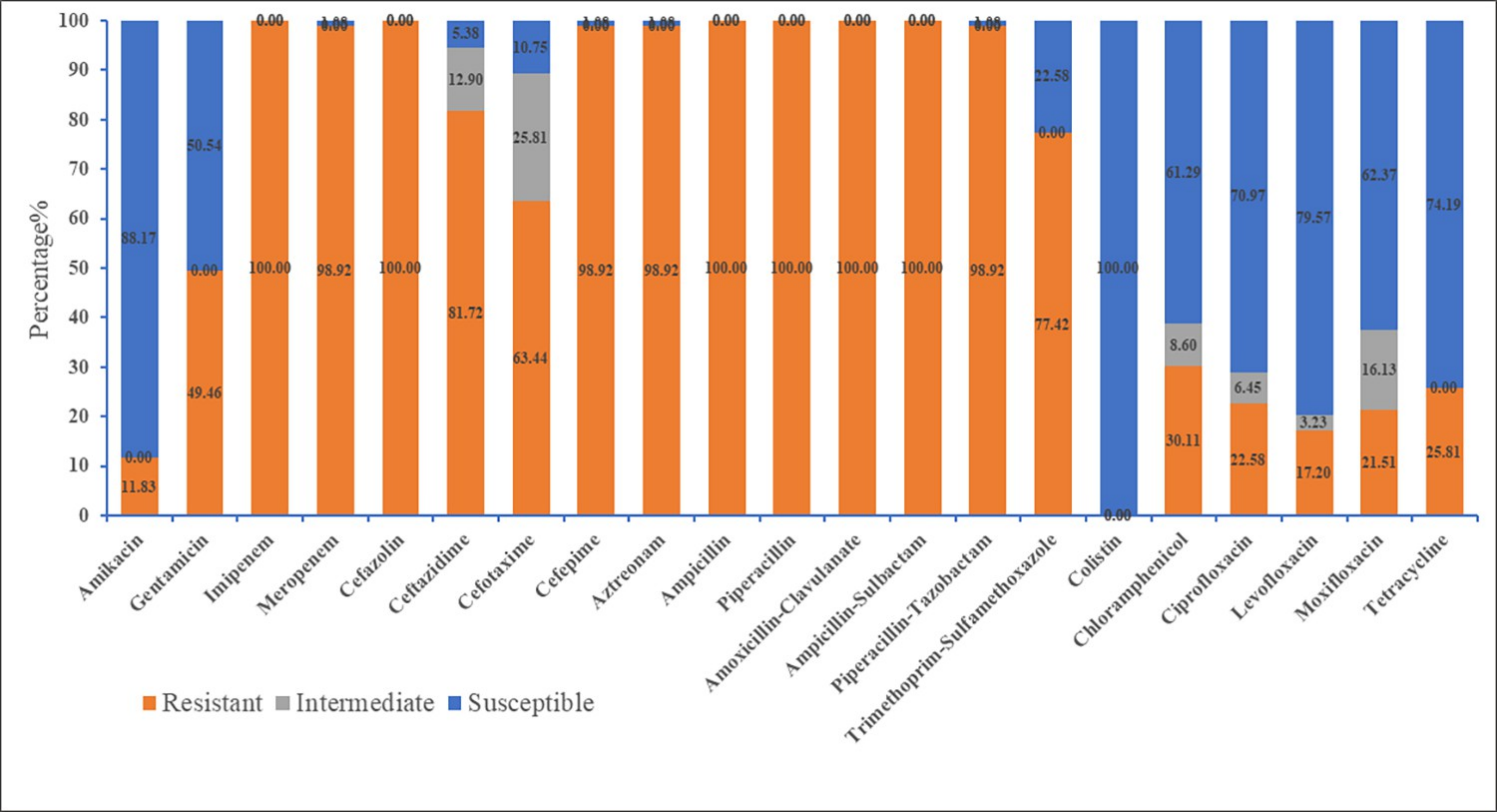
Resistance rates of *Klebsiella pneumoniae* isolates to 21 antibacterial drugs.

The MIC values of imipenem in the 93 CRKP isolates were determined, with 2 (2.15%) at 256 μg/ml, 18 (19.35%) at 128 μg/ml, 21 (22.58%) at 64 μg/ml, 22 (23.66%) at 32 μg/ml, 15 (16.13%) at 16 μg/ml, and 15 (16.13%) at 8 μg/ml (Fig 2). Furthermore, we performed susceptibility testing for 21 antibiotics and all isolates showed reduced susceptibility to at least 10 or more antibiotics. The resistance rates to imipenem and meropenem were 100% and 98.92%, respectively. The resistance rates to cefazolin, ceftazidime, cefotaxime, cefepime, aztreonam, piperacillin–tazobactam were 100%, 81.72%, 63.44%, 98.92%, 98.92%, and 98.92%, respectively. All of the CRKP isolates were resistant to ampicillin, piperacillin, amoxicillin–clavulanate, and ampicillin–sulbactam. The resistance rates to trimethoprim–sulfamethoxazole, chloramphenicol, ciprofloxacin, levofloxacin, moxifloxacin and tetracycline were 77.42%, 30.11%, 22.58%, 17.20%, 21.51% and 25.81%, respectively (S2 Table). All CRKP isolates were sensitive to colistin (Fig 3). Among the isolates, 32 different resistance patterns were observed. It was worth noting that 77 (82.80%) were MDR isolates, and 16 (17.20%) were XDR isolates. The most (18.28%, 17/93) common MDR pattern was IMP-MEM-CFZ-CAZ-CTX-FEP-AZT-AMP-PIP-AMC-SAM-TZP-SXT. Among the 13 XDR CRKP isolates, 10 (76.92%) isolates had the same drug resistance pattern (AMK-GEN-IMP-MEM-CFZ-CAZ-CTX-FEP-AZT-AMP-PIP-AMC-SAM-TZP-SXT-CIP-LVX-MXF-TET) and 2 (15.38%) isolates were only sensitive to amikacin, with a resistance pattern (GEN-IMP-MEM-CFZ-CAZ-CTX-FEP-AZT-AMP-PIP-AMC-SAM-TZP-SXT-CIP-LVX-MXF-TET) (Table 1). Only one (7.69%) strain was resistant to all tested antibiotics except colistin.

**Table 1:** 32 drug resistance patterns in the 93 *Klebsiella Pneumoniae* isolates in this study.

No.	Resistance patterns	No. of strains
1	IMP MEM CFZ FEP AZT AMP PIP AMC SAM TZP	6
2	GEN IMP CFZ CAZ AMP PIP AMC SAM SXT CHL TET	1
3	IMP MEM CFZ FEP AZT AMP PIP AMC SAM TZP SXT	2
4	IMP MEM CFZ CAZ FEP AZT AMP PIP AMC SAM TZP	2
5	GEN IMP MEM CFZ FEP AZT AMP PIP AMC SAM TZP	2
6	IMP MEM CFZ CAZ FEP AZT AMP PIP AMC SAM TZP SXT	3
7	GEN IMP MEM CFZ FEP AZT AMP PIP AMC SAM TZP SXT	1
8	IMP MEM CFZ CAZ CTX FEP AZT AMP PIP AMC SAM TZP	5
9	GEN IMP MEM CFZ CAZ FEP AZT AMP PIP AMC SAM TZP	1
10	GEN IMP MEM CFZ CAZ FEP AZT AMP PIP AMC SAM TZP SXT	4
11	IMP MEM CFZ CAZ CTX FEP AZT AMP PIP AMC SAM TZP SXT	17
12	GEN IMP MEM CFZ CTX FEP AZT AMP PIP AMC SAM TZP CHL	1
13	GEN IMP MEM CFZ FEP AZT AMP PIP AMC SAM TZP SXT CHL	3
14	GEN IMP MEM CFZ FEP AZT AMP PIP AMC SAM TZP SXT CIP	1
15	IMP MEM CFZ CAZ FEP AZT AMP PIP AMC SAM TZP SXT CHL	3
16	GEN IMP MEM CFZ CAZ CTX FEP AZT AMP PIP AMC SAM TZP	1
17	IMP MEM CFZ CAZ CTX FEP AZT AMP PIP AMC SAM TZP CHL TET	1
18	GEN IMP MEM CFZ CAZ FEP AZT AMP PIP AMC SAM TZP SXT CHL	5
19	IMP MEM CFZ CAZ CTX FEP AZT AMP PIP AMC SAM TZP SXT CHL	3
20	IMP MEM CFZ CAZ CTX FEP AZT AMP PIP AMC SAM TZP SXT TET	1
21	GEN IMP MEM CFZ CAZ CTX FEP AZT AMP PIP AMC SAM TZP SXT	4
22	IMP MEM CFZ CAZ CTX FEP AZT AMP PIP AMC SAM TZP SXT MXF TET	1
23	GEN IMP MEM CFZ CAZ CTX FEP AZT AMP PIP AMC SAM TZP CHL TET	1
24	GEN IMP MEM CFZ CAZ CTX FEP AZT AMP PIP AMC SAM TZP CIP MXF	1
25	GEN IMP MEM CFZ CAZ CTX FEP AZT AMP PIP AMC SAM TZP SXT CHL	4
26	GEN IMP MEM CFZ CAZ CTX FEP AZT AMP PIP AMC SAM TZP SXT CHL CIP TET	1
27	GEN IMP MEM CFZ CTX FEP AZT AMP PIP AMC SAM TZP SXT CHL CIP MXF TET	1
28	GEN IMP MEM CFZ CAZ CTX FEP AZT AMP PIP AMC SAM TZP SXT CIP MXF TET	1
29	IMP MEM CFZ CAZ CTX FEP AZT AMP PIP AMC SAM TZP SXT CHL CIP LVX MXF TET	3
30	GEN IMP MEM CFZ CAZ CTX FEP AZT AMP PIP AMC SAM TZP SXT CIP LVX MXF TET	2
31	AMK GEN IMP MEM CFZ CAZ CTX FEP AZT AMP PIP AMC SAM TZP SXT CIP LVX MXF TET	10
32	AMK GEN IMP MEM CFZ CAZ CTX FEP AZT AMP PIP AMC SAM TZP SXT CHL CIP LVX MXF TET	1

Abbreviations: amikacin, AMK; gentamicin, GEN; impenem, IMP; meropenem, MEM; cefazolin, CFZ; ceftazidime, CAZ; cefotaxime, CTX; cefepime, FEP; amtrinan, ATM; ampicillin, AMP; piperacillin, PIP; amoxicillin/clavulanic, AMC; ampicillin/Sulbactam, SAM; piperacillin/tazobactam, TZP; colistin, CST; trimethoprim/sulfamethoxazole, SXT; chloramphenicol, CHL; ciprofloxacin, CIP; levofloxacin, LVX; moxifloxacin, MXF; tetracycline, TET.

### Multi-locus sequence typing of the isolated CRKP

Multiple site sequencing was performed on 93 isolates of CRKP, of which 55 isolates detected ST type. Among the isolates, 55 strains were detected with ST type, of which ST 11 type 12.9% (12/93) and ST 197 type 12.9% (12/93) were the main ST types. Other detailed results are shown in the S3 Table.

### Antibiotic resistance genes and integrons of the isolated CRKP

**Fig 4:**
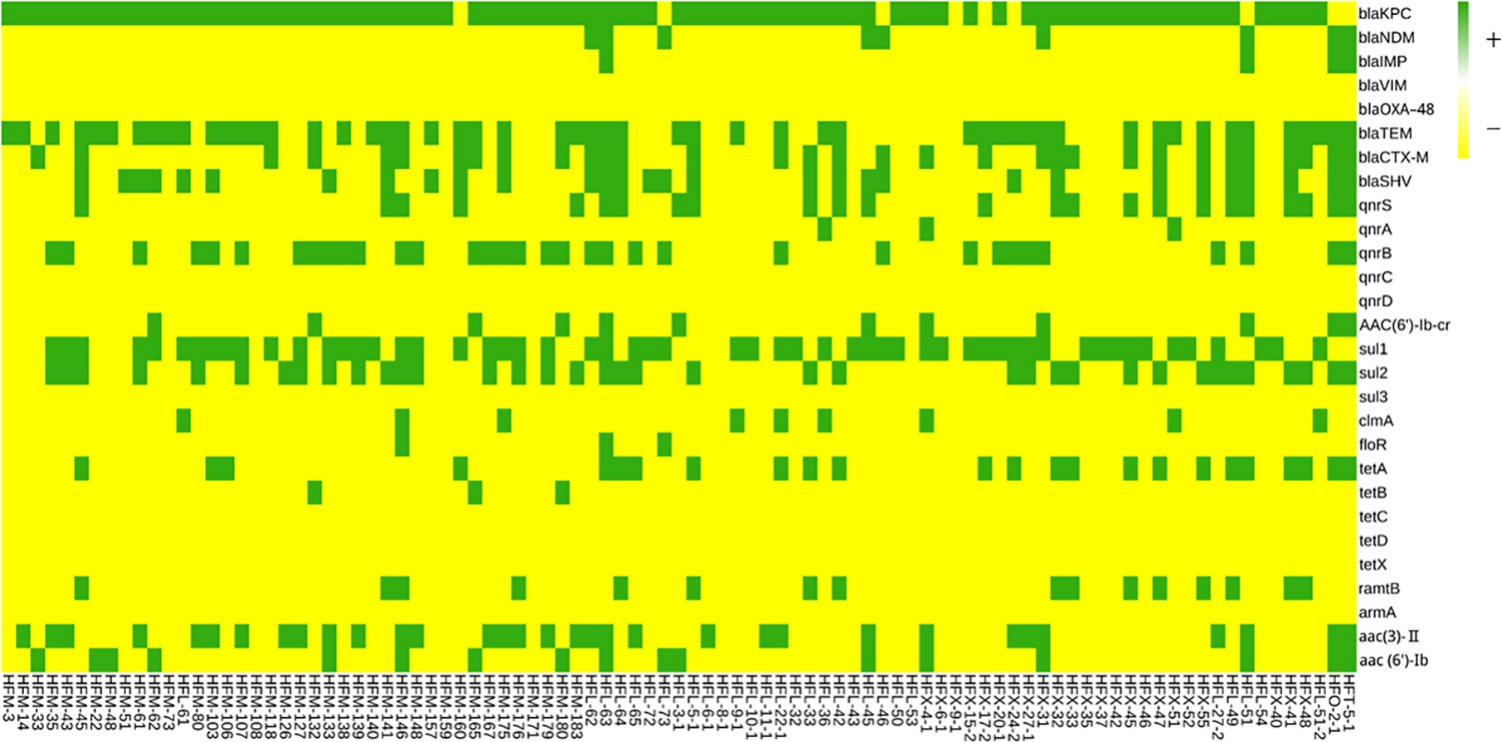
The heat map of drug resistance gene detection in the 93 *Klebsiella Pneumoniae* isolates in this study.

Three carbapenemase resistance genes, *bla*_KPC_, *bla*_NDM_, and *bla*_IMP_, were detected. The *bla*_KPC_ was carried by 85 (91.4%) isolates, *bla*_NDM_ by 9 (9.68%) isolates, and *bla*_IMP_ by 4 (4.3%) isolates. No isolates carried *bla*_VIM_ or *bla*_OXA-48_. ESBLs genes were also detected, including 32 (34.4%) carrying *bla*_CTX-M_, 30 (32.26%) carrying *bla*_SHV_, and 53 (56.99%) carrying *bla*_TEM_. The detection rates of the fluoroquinolone resistance genes *qnrA*, *qnrB*, *qnrS*, and *aac(6’)-Ib-cr* were 3.22% (3/93), 36.56% (34/93), 25.8% (25/93) and 12.9% (12/93), respectively, *qnrC* and *qnrD* were not detected. The detection rates of the sulfonamide resistance genes *sul1* and *sul2* were 60.22% (56/93) and 39.78% (37/93), respectively, *sul3* was not detected. The detection rates of the chloramphenicol resistance genes *cmlA* and *floR* were 9.68% (9/93) and 3.22% (3/93), respectively. The detection rates of the tetracycline resistance genes *tet(A)* and *tet(B)* were 25.8% (24/93) and 3.23% (3/93), respectively. No *tet(C)*, *tet(D)* and *tet(X)* genes were detected. The detection rates of the aminoglycoside resistance genes *rmtB*, *aac(3)-II*, and *aac(6′)-Ib* were 13.98% (13/93), 35.48% (33/93), 18.28% (17/93), respectively. *ArmA*, *rmtB* and *rmtC* were not detected (Fig 4). Only class 1 integron was detected in 72 (61.0%) of the 93 CRKP isolates. The outer membrane pore protein genes were analyzed; 3 (3.23%) isolates did not carry OmpK-35, and 2 (2.15%) isolates did not carry OmpK36.

### Detection of virulence genes of the isolated CRKP

Four virulence genes were detected in 11 of the 93 isolates. 6 (6.45%) isolates carried *rmpA*, and 11 (11.83%) isolates carried *rmpA2*. The iron uptake system genes *iucA*, *iroB* were both carried by 10 isolates, accounting for 10.75% of the isolates. Importantly, 6 isolates carried all four virulence genes, accounting for 6.45% of the isolates. Among these 11 isolates, 1 was an MDR isolates, and the others 10 isolates were MDR isolates (S4 Table). These 11 CRKP isolates carrying virulence genes were all ST11.

### Various replicon types of the isolated CRKP

The results of PBRT revealed 12 replicon types. 44 (47.31%) isolates harbored two replicons, and 24 (25.8%) harbored one replicon. Twelve (12.9%) isolates harbored three replicons, 2 (2.15%) harbored four replicons, and 3 (3.23%) harbored five replicons. One isolate harbored six replicons. The most common replicon type was IncFII, which was harbored by 50 (53.76%) isolates, followed by IncR and IncIB, harbored by 35 (37.63%) and 31 (33.33%) isolates, respectively. No replicon types were found in 7 (7.53%) isolates. Among the isolates, the most common type of replicon combination was IncFII and IncR, accounting for 11 (11.83%), followed by the combination of IncFIB and IncFIA, accounting for 10 (10.75%) (S5 Table).

### Disinfectant resistance testing and biofilm formation of the isolated CRKP

All isolates were resistant to disinfectant. The NaClO MIC value of 67 (72.04%) isolates was 400 μg/ml, and that of the other isolates was 800 μg/ml. Regarding biofilm formation, 63 (67.74%) isolates exhibited biofilm formation capabilities; among them, 58 (63.4%) isolates were weak film-forming, and 5 (5.4%) were moderate. The other 30 (31.2%) isolates were unable to form biofilms (S6 Table). Among the 63 biofilm producers, 14 (22.22%) were XDR and 49 (77.78%) were MDR. Among the 30 CRKPs that could not form biofilms, 2 (6.67%) were XDR, and 28 (93.33%) were MDR. Regarding drug resistance among CRKP isolates, the biofilm-forming ability of CRKP was significantly different from the resistance of different classes of antibiotics(p = 0.019).

## Discussion

The source of hospital sewage is very complex, including medical and domestic sewage. Hospital sewage contains highly resistant microorganisms and is a suitable niche for bacterial horizontal gene transfer. We conducted this study to understand the characteristics of highly resistant bacteria in hospital sewage.

CRKP was the most common species of carbapenem-resistant bacteria isolated from the sewage samples. This finding is probably because the temperature in the Northern Hemisphere begins to increase in May, and July to September are the hottest months of the year; *K*. *pneumoniae* survives better at higher temperatures and humidity levels, and the incidence of KP bloodstream infections varies seasonally [16], this is also consistent with our research.

Antibiotic resistance has always been a key and difficult issue of global concern, and hospital sewage is a key reservoir for MDR microorganisms. Except for β lactam antibiotics, CRKP isolates also have different degrees of resistance to other antibiotics, and the antimicrobial spectrum is complex.

In addition, the biofilm formation ability of CRKP isolates not only makes it easier for these isolates to colonize in the sewers [17], but also shows significant differences in antibiotic resistance. And regarding the drug resistance pattern, biofilm-producing isolates were more resistant than non-biofilm-producing isolates, consistent with previous reports [11]. Biofilm-forming CRKP is more dangerous because these isolates can colonize the respiratory, gastrointestinal, and urinary tracts and can lead to invasive infections (especially in immune-deficient patients) [18]. With the increasing prevalence of multidrug-resistant KP, effective management of infections caused by biofilm-producing microorganisms is essential in healthcare settings.

In addition, all of these CRKP isolates were resistant to chlorine-containing disinfectants, potentially because the sewage contained disinfectants from the operating rooms and wards that had been diluted to sublethal concentration by domestic sewage and other water; bacteria living in this sublethal concentration environment might develop resistance [19]. Furthermore, it has been reported that bacteria with biofilm growth patterns can develop resistance to disinfectants [20]. Further research is required to explain why these KP isolates survive in sewage. Fortunately, when we tested the samples from the outlet, no live bacteria were detected, indicating that the sewage digestion tank eliminated these isolates.

The MIC value of imipenem for most CRKP isolates is 32 μg/mL and 64 μg/mL, and *bla*_*KPC*_ is the most common carbapenem resistant gene in this study. In the national surveillance report of Chinese clinical carbapenem-resistant *Enterobacteriaceae* (CRE) strains isolated from 2012 to 2016, the detection rate of *bla*_KPC_ was 77.1%, that of *bla*_NDM_ was 11.3%, and that of *bla*_IMP_ was 1.8% [21]. However, in north China, the incidence of *bla*_KPC_ was higher than the average level, reaching 90.32%, a detection rate consistent with our results [22, 23]. Among the 93 isolates, 4 CRKP isolates did not carry any carbapenemase genes. The MIC value was 8 μg/mL for imipenem in two of these isolates and 16 μg/mL in the other two, both representing low carbapenem resistance levels. This finding may be due to other rare carbapenem resistance genes; ESBL and/or AmpC production and changes in the cell membrane may also be responsible [24, 25]. ESBLs and AmpCs can hydrolyze carbapenems at very low levels; when combined with reduced membrane impermeability or increased drug efflux, these strains can demonstrate resistance. Notably, in the present study, four CRKP isolates without deletion of Ompk-35 or Ompk-36 protein were observed. We hypothesize that, in addition to Ompk-35, Ompk-36 outer membrane pore protein deletion, the deletion of other outer membrane pore proteins, or alterations in the exopump system may be involved in conferring increased resistance.

Plasmids play an important role in spreading antibiotic resistance [26]. In our study, IncFIB and IncFII were dominate replicons, and these types of plasmids were also popular vectors for carbapenem resistance genes such as *bla*_NDM_, *bla*_KPC_, and *ESBL genes bla*_CTX–M_, and *bla*_OXA_ [27, 28]. The wide variety of replicon types increases drug-resistant gene transmission and the genetic evolution of drug-resistant bacteria.

Among highly drug-resistant isolates of CRKP, ST11 was the most common sequence type, which is characterized by high virulence and high drug resistance [29]. ST11 is one of the most important hospital-acquired carbapenem-resistant *K*. *pneumoniae* (HA-CRKP) clones [30]. In our study, all 11 ST11 XDR isolates harbored the HVKP -related molecular marker genes *iroB*, *iucA*, *rmpA*, and *rmpA2* [11]. Research has shown an association between *rmpA* and purulent liver abscesses [31]; overproduction of capsule regulator genes (*rmpA* or *rmpA2*), salmochelin (*iroBCDN*) and aerocin (*iucABCD*) siderophore gene clusters leads to higher virulence, as shown in a neutrophil killing assay (NKA) and in *Galleria mellonella* infection model infection assays [32]. These virulence genes have been suggested to be present in virulence plasmids [33]. ST11-type CR-HVKP has been reported to cause severe infection in relatively healthy people in China due to the acquisition of the virulence plasmid pLVPK, making infection difficult to treat with antibiotics [32]. Hybrid-binding virulence plasmids have been shown to be transferable by conjugation to other CRKP isolates, transforming ST11 CRKP strains into Hv-CRKP strains [4]. Thus, the ST11 CRKP isolates in this study had high drug resistance and the potential for high virulence and transmission.

## Conclusions

In this study, CRKP was the most Carbapenem drug-resistant bacteria isolated from hospital sewage, the resistance of some antibiotics may be related to the ability of biofilm formation. CRKP isolates were all tolerant to chlorine-containing disinfectants, which may be related to disinfectants used in the environment for a long time. In this study, *bla*_KPC_ was the major Carbapenem gene, and some virulence genes were also detected. The Multilocus sequence typing of CRKP isolates were diverse, and ST11 was closely related to hv-CRKP. Although we examined the replication subtype in CRKP isolates, however, further studies are needed to clarify whether the resistance and virulence characteristics of these isolates are influenced by mobile genetic factors, such as plasmids, and whether they pose a risk of transmission. Improper treatment of hospital sewage may lead to the spread of drug-resistant bacteria and antibiotic-resistant genes, which may become a substantial threat to public health.

## Supporting information

S1 Table PCR primers and conditions. (DOCX)

S2 Table Antibiotic resistance of CRKP isolates. (XLSX)

S3 Table Multiple site sequence typing of CRKP isolates. (XLSX)

S4 Table Detailed information of 11 CRKP isolates with virulence genes in this study, including virulence genes, MLST results, MIC values of imipenem, and antibiotic resistance. (XLSX)

S5 Table Replicon typing in CRKP isolates. (XLSX)

S6 Table Biofilm formation capacity in CRKP isolates. (XLSX)
