# Supplementary material for: Characteristics of Carbapenem-resistant Klebsiella pneumoniae in sewage from a tertiary hospital in Jilin Province, China
Source: PLoS One. 2023 May 17;18(5):e0285730. doi: 10.1371/journal.pone.0285730 (PMC10191347; doi:10.1371/journal.pone.0285730)
Supplement: S1 Table — (DOCX) [file pone.0285730.s001.docx]

**Table S3** Primers of AMR genes in this study

| Gene type | Primes^a^ | | Sequence (5’-3’) | AL^b^ (bp) | AT^c^ (℃) | Refence |
| --- | --- | --- | --- | --- | --- | --- |
| ESBLs | *bla*_CTX-M_ | F | ACGTTGCATATATCGACGTTG | 544 | 54 | (Xia and Li et al., 2020) |
|  |  | R | GCAGCGACTGTCGTACCCTAT |  |  |  |
|  | *bla*_SHV_ | F | CGCCGGGTTATTCTTATTTGTCGC | 1017 | 55 | (Kong and Cai et al., 2019) |
|  |  | R | TCTTTCCGATGCCGCCGCCAGTCA |  |  |  |
|  | *bla*_TEM_ | F | TCGCCGCATACACTATTCTCAGAATGA | 445 | 55 | (Ballén and Gabasa et al., 2021) |
|  |  | R | ACGCTCACCGGCTCCAGATTTAT |  |  |  |
| Carbapenem resistance gene | *IMP* | F | GGAATAGAGTGGCTTAAYTCTC | 232 | 52 | (Hatrongjit and Kerdsin et al., 2018) |
|  |  | R | GGTTTAAYAAAACAACCACC |  |  |  |
|  | *VIM* | F | GCMCTTCTCGCGGAGATTGA | 257 | 55 | (Kong and Cai et al., 2019) |
|  |  | R | TGCGCAGCACCRGGATAGA |  |  |  |
|  | *OXA-48* | F | GCGTGGTTAAGGATGAACAC | 438 | 52 | (Hatrongjit and Kerdsin et al., 2018) |
|  |  | R | CATCAAGTTCAACCCAACCG |  |  |  |
|  | *NDM* | F | TCGCCCCATATTTTTGCTAC | 1080 | 56.5 | (Feng and Liu et al., 2019) |
|  |  | R | CTGGGTCGAGGTCAGGATAG |  |  |  |
|  | *KPC* | F | ATGTCACTGTATCGCCGTCT | 893 | 57.8 | (Cejas and Fernández et al., 2014) |
|  |  | R | TTTTCAGAGCCTTACTGCCC |  |  |  |
| Aminoglycoside resistance gene | *armA* | F | ATGGATAAGAATGATGTTGTTAAG | 774 | 55 | (Wang and Chen et al., 2022) |
|  |  | R | TTATTTCTGAAATCCACTAGTAATTA |  |  |  |
|  | *rmtB* | F | ACATCAACGATGCCCTCAC | 724 | 54 | (Yu and Zhang et al., 2021) |
|  |  | R | AAGTTCTGTTCCGATGGTC |  |  |  |
|  | *aac(3)-II* | F | ACTGTGATGGGATACGCGTC | 237 | 55 | (Wang and Chen et al., 2022) |
|  |  | R | CTCCGTCAGCGTTTCAGCTA |  |  |  |
|  | *aac(6′)-Ib* | F | ATGACTGAGCATGACCTTGC | 519 | 52 | (Rasoulinasab and Shahcheraghi et al., 2021) |
|  |  | R | TTAGGCATCACTGCGTGTTC |  |  |  |
| Quinolone resistance gene | *aac(6′)-Ib-cr* | F | ATATGCGGATCCAAAAACAAAGTTAGGCATCACAAAG | 810 | 61.5 | (Warburg and Korem et al., 2009) |
|  |  | R | ATATGCGAATTCCTTCAGTTCCTTCAAATAATGGAGA |  |  |  |
|  | *qnrA* | F | ATTTCTTCACGCCAGGATTTG | 516 | 53 | (Yu and Zhang et al., 2021) |
|  |  | R | GATCGGCAAAGGTTAGGTCA |  |  |  |
|  | *qnrB* | F | GATCGTGAAAGCCAGAAAGG | 469 | 53 | (Yu and Zhang et al., 2021) |
|  |  | R | ACGATGCCTGGTAGTTGTCC |  |  |  |
|  | *qnrC* | F | GGGTTGTACATTTATTGAATCG | 307 | 47 | (Saki and Farajzadeh et al., 2022) |
|  |  | R | CACCTACCCATTTATTTTCA |  |  |  |
|  | *qnrD* | F | CGAGATCAATTTACGGGGAATA | 533 | 50 | (Saki and Farajzadeh et al., 2022) |
|  |  | R | AACAAGCTGAAGCGCCTG |  |  |  |
|  | *qnrS* | F | ACGACATTCGTCAACTGCAA | 417 | 53 | (Yu and Zhang et al., 2021) |
|  |  | R | TAAATTGGCACCCTGTAGGC |  |  |  |
| Tetracycline resistance gene | *tetA* | F | GTAATTCTGAGCACTGTCGC | 737 | 62 | (Yuan and Liang et al., 2021) |
|  |  | R | CTGCCTGGACAACATTGCTT |  |  |  |
|  | *tetB* | F | CTCAGTATTCCAAGCCTTTG | 416 | 57 | (Yuan and Liang et al., 2021) |
|  |  | R | ACTCCCCTGAGCTTGAGGGG |  |  |  |
|  | *tetC* | F | CTTGAGAGCCTTCAACCCAG | 418 | 60 | (Jain and Bepari et al., 2021) |
|  |  | R | ATGGTCGTCATCTACCTGAG |  |  |  |
|  | *tetD* | F | AAACCATTACGGCATTCTGC | 787 | 62 | (Yuan and Liang et al., 2021) |
|  |  | R | GACCGGATACACCATCCATC |  |  |  |
|  | *tetX* | F | GAAAGAGACAACGACCGAGAG | 131 | 57 | (Chen and Zhao et al., 2022) |
|  |  | R | ACACCCATTGGTAAGGCTAAG |  |  |  |
| Sulfonamide resistance gene | *Sul1* | F | CACCGGAAACATCGCTGCA | 158 | 58 | (Yuan and Liang et al., 2021) |
|  |  | R | AAGTTCCGCCGCAAGGCT |  |  |  |
|  | *Sul2* | F | CTCCGATGGAGGCCGGTAT | 190 | 58 | (Yuan and Liang et al., 2021) |
|  |  | R | GGGAATGCCATCTGCCTTGA |  |  |  |
|  | *Sul3* | F | CCCATACCCGGATCAAGAATAA | 143 | 57 | (Yuan and Liang et al., 2021) |
|  |  | R | CAGCGAATTGGTGCAGCTACTA |  |  |  |
| Phenicol resistance gene  outer membrane porins genes | *floR* | F | GGCTTTCGTCATTGCGTCTC | 650 | 55 | (Yuan and Liang et al., 2021) |
|  |  | R | ATCGGTAGGATGAAGGTGAGGA |  |  |  |
|  | *cmlA* | F | TGCCAGCAGTGCCGTTTAT | 900 | 55 | (Yuan and Liang et al., 2021) |
|  |  | R | ATCGGTAGGATGAAGGTGAGGA |  |  |  |
|  | *OmpK-35* | F | ATGATGAAGCGCAATATTCTGGCAGTGG | 650 | 55 | (El-Domany and Awadalla et al., 2021) |
|  |  | R | TCGGCTTTGTCGCCATTGCCGTCA |  |  |  |
|  | *OmpK-36* | F | ATGAAAGTTAAAGTACTGTCCCTC | 900 | 55 | (El-Domany and Awadalla et al., 2021) |
|  |  | R | GCCGGTATCTCTACCGACGAC |  |  |  |
|  |  |  |  |  |  |  |

**Table S3** Primers of integrase genes in this study

| Gene type | Primes^a^ | | Sequence (5’-3’) | AL^b^ (bp) | AT^c^ (℃) | Refence |
| --- | --- | --- | --- | --- | --- | --- |
| integrase genes | *intI1* | F | ACATGTGATGGCGACGCACGA | 569 | 55 | (Cui and Wang et al., 2015) |
|  |  | R | ATTTCTGTCCTGGCTGGCGA |  |  |  |
|  | *intI2* | F | GTGCAACGCATTTTGCAGG | 403 | 55 | (Yuan and Liang et al., 2021) |
|  |  | R | CAACGGAGTCATGCAGATG |  |  |  |
|  | *intI3* | F | CATTTGTGTTGTGGACGGC | 717 | 55 | (Yuan and Liang et al., 2021) |
|  |  | R | GACAGATACGTGTTTGGCAA |  |  |  |
|  |  |  |  |  |  |  |

**Table S3** Primers of virulence genes in this study

| Gene type | Primes^a^ | | Sequence (5’-3’) | AL^b^ (bp) | AT^c^ (℃) | Refence |
| --- | --- | --- | --- | --- | --- | --- |
| integrase genes | *iucA* | F | ACTGGGCTACCTCTGCTTCA | 2979 | 58 | (Xu and Fu et al., 2019) |
|  |  | R | TAACGGCGATAAACCTCG |  |  |  |
|  | *IroB* | F | ATCTCATCATCTACCCTCCGCTC | 235 | 59 | (Russo and Olson et al., 2018) |
|  |  | R | GGTTCGCCGTCGTTTTCAA |  |  |  |
|  | *rmpA* | F | ACTGGGCTACCTCTGCTTCA | 516 | 58 | (Cejas and Fernández et al., 2014) |
|  |  | R | CTTGCATGAGCCATCTTTCA |  |  |  |
|  | *rmpA2* | F | TGTGCAATAAGGATGTTACATTAGT | 609 | 56 | (Xu and Fu et al., 2019) |
|  |  | R | TTTGATGTGCACCATTTTTCA |  |  |  |
|  |  |  |  |  |  |  |

**References:**

Ballén, V. and Y. Gabasa, et al. (2021). "Correlation Between Antimicrobial Resistance, Virulence Determinants and Biofilm Formation Ability Among Extraintestinal Pathogenic Escherichia coli Strains Isolated in Catalonia, Spain." Front Microbiol **12**: 803862.

Cejas, D. and C. L. Fernández, et al. (2014). "First isolate of KPC-2-producing Klebsiella pneumonaie sequence type 23 from the Americas." J Clin Microbiol **52**(9): 3483-5.

Chen, T. and M. Zhao, et al. (2022). "Serious Risk of Tigecycline Resistance in Escherichia coli Isolated from Swine Manure." Microb Ecol.

Cui, X. and J. Wang, et al. (2015). "Prevalence and antimicrobial resistance of Shigella flexneri serotype 2 variant in China." Front Microbiol **6**: 435.

El-Domany, R. A. and O. A. Awadalla, et al. (2021). "Analysis of the Correlation Between Antibiotic Resistance Patterns and Virulence Determinants in Pathogenic Klebsiella pneumoniae Isolates from Egypt." Microb Drug Resist **27**(6): 727-739.

Feng, Y. and L. Liu, et al. (2019). "Key evolutionary events in the emergence of a globally disseminated, carbapenem resistant clone in the Escherichia coli ST410 lineage." Commun Biol **2**: 322.

Hatrongjit, R. and A. Kerdsin, et al. (2018). "Detection of plasmid-mediated colistin-resistant and carbapenem-resistant genes by multiplex PCR." MethodsX **5**: 532-536.

Jain, P. and A. K. Bepari, et al. (2021). "High prevalence of multiple antibiotic resistance in clinical E. coli isolates from Bangladesh and prediction of molecular resistance determinants using WGS of an XDR isolate." Sci Rep **11**(1): 22859.

Kong, Z. and R. Cai, et al. (2019). "First Reported Nosocomial Outbreak Of NDM-5-Producing Klebsiella pneumoniae In A Neonatal Unit In China." Infect Drug Resist **12**: 3557-3566.

Rasoulinasab, M. and F. Shahcheraghi, et al. (2021). "Distribution of ciprofloxacin-resistance genes among ST131 and non-ST131 clones of Escherichia coli isolates with ESBL phenotypes isolated from women with urinary tract infection." Iran J Microbiol **13**(3): 294-302.

Russo, T. A. and R. Olson, et al. (2018). "Identification of Biomarkers for Differentiation of Hypervirulent Klebsiella pneumoniae from Classical K. pneumoniae." J Clin Microbiol **56**(9).

Saki, M. and S. A. Farajzadeh, et al. (2022). "Occurrence of plasmid-mediated quinolone resistance genes in Pseudomonas aeruginosa strains isolated from clinical specimens in southwest Iran: a multicentral study." Sci Rep **12**(1): 2296.

Wang, N. and X. Chen, et al. (2022). "Artemisinin derivative DHA27 enhances the antibacterial effect of aminoglycosides against Pseudomonas aeruginosa by inhibiting mRNA expression of aminoglycoside-modifying enzymes." Front Pharmacol **13**: 970400.

Warburg, G. and M. Korem, et al. (2009). "Changes in aac(6')-Ib-cr prevalence and fluoroquinolone resistance in nosocomial isolates of Escherichia coli collected from 1991 through 2005." Antimicrob Agents Chemother **53**(3): 1268-70.

Xia, Y. and H. Li, et al. (2020). "Antimicrobial Drug Resistance in Salmonella enteritidis Isolated From Edible Snakes With Pneumonia and Its Pathogenicity in Chickens." Front Vet Sci **7**: 463.

Xu, M. and Y. Fu, et al. (2019). "High prevalence of KPC-2-producing hypervirulent Klebsiella pneumoniae causing meningitis in Eastern China." Infect Drug Resist **12**: 641-653.

Yu, B. and Y. Zhang, et al. (2021). "Analysis of antibiotic resistance phenotypes and genes of Escherichia coli from healthy swine in Guizhou, China." Onderstepoort J Vet Res **88**(1): e1-e8.

Yuan, Y. and B. Liang, et al. (2021). "Migratory wild birds carrying multidrug-resistant Escherichia coli as potential transmitters of antimicrobial resistance in China." PLoS One **16**(12): e0261444.
